# Resistance Allele Frequency of *Helicoverpa zea* to Vip3Aa *Bacillus thuringiensis* Protein in the Southeastern U.S.

**DOI:** 10.3390/insects14020161

**Published:** 2023-02-07

**Authors:** José C. Santiago-González, David L. Kerns, Fei Yang

**Affiliations:** Department of Entomology, Texas A&M University, College Station, TX 77843-2475, USA

**Keywords:** *Bacillus thuringiensis*, *Helicoverpa zea*, Vip3Aa, resistance, allele

## Abstract

**Simple Summary:**

Evolution of insect resistance is the main threat to the sustainability of the Bt technology. *Helicoverpa zea* (Boddie) (Lepidoptera: Noctuidae) is a major target pest of Bt corn and cotton. The purpose of this study is to determine the frequency of alleles conferring resistance to Vip3Aa in *H. zea* in the mid-south region of the U.S. Using a modified F_2_ screen method, we found the major resistance allele frequency for Vip3Aa in *H. zea* collected from four southern states during 2019–2020 was 0.0155, suggesting that the frequency of Vip3Aa resistance alleles in *H. zea* is not rare in the field.

**Abstract:**

*Helicoverpa zea* is a major target pest of Bt crops expressing Cry and/or Vip3Aa proteins in the U.S.A. Widespread practical resistance of *H. zea* to the Cry1 and Cry2 proteins makes Vip3Aa the only effective Bt protein against this pest. Understanding the frequency of resistance alleles against Vip3Aa in field populations of *H. zea* is crucial for resistance management and the sustainability of Vip3Aa technology. Using a modified F_2_ screen method by crossing susceptible laboratory female moth with feral male moth of *H. zea*, we successfully screened a total of 24,576 neonates from 192 F_2_ families of *H. zea* collected from Arkansas, Louisiana, Mississippi, and Tennessee during 2019–2020. We found five F_2_ families containing ≥3rd instar survivors on the diagnostic concentration of 3.0 µg/cm^2^ Vip3Aa39. Dose-response bioassays confirmed the high levels of Vip3Aa resistance in these F_2_ families, with an estimated resistance ratio of >909.1-fold relative to the susceptible strain. The estimated resistance allele frequency against Vip3Aa in *H. zea* for these four southern states is 0.0155 with a 95% CI of 0.0057–0.0297. These data should provide critical information for understanding the risks of Vip3Aa resistance in *H. zea* and help design appropriate resistance management strategies for the sustainability of the Vip3Aa technology.

## 1. Introduction

Crops genetically engineered to produce the Cry and Vip insecticidal proteins from the soil-inhabiting bacteria *Bacillus thuringiensis* (Bt) have been widely adopted for control of some pestiferous insect pests. Relative to the conventional insecticides, the Bt technology has greatly increased pest control efficacy due to its continuous and constitutive expression in genetically modified crops as disruptors of insect midgut membranes [1,2,3,4,5]. Large-scale adoption of Bt crops in the U.S. has resulted in significant reductions in insecticide use, high profits for growers [6,7], and reduced risks for human health and the environment [8]. However, extensive and continuous use of Bt crops has placed high selection pressure for Bt resistance on several insect species, diminishing their efficacy [9]. To prolong the lifespan of these Bt technologies, government agencies in the U.S. have adopted two main insect resistance management (IRM) strategies, known as the high-dose/refuge and pyramid/refuge, to delay the evolution of insect resistance.

*Helicoverpa zea* (Boddie) (Lepidoptera: Noctuidae) is a detrimental pest of many economically important crops and a major target species of Bt technologies. In the U.S., the Bt proteins used in corn and cotton plants for managing *H. zea* include Cry1Ab, Cry1Ac, Cry1F, Cry1A.105, Cry2Ab, Cry2Ae, and Vip3Aa. Many previous studies have reported that *H. zea* has developed widespread practical resistance to the Cry1 and Cry2 proteins produced in Bt cotton and Bt corn in the U.S. [10,11,12,13]. By contrast, no practical resistance has been reported to the Vip3Aa corn and cotton for *H. zea*. Field efficacy data also showed that Vip3Aa technology could provide exceptional protection against *H. zea* damage [14]. However, purified protein bioassays from 2016–2020 in the southern U.S. showed a small but significant decrease in susceptibility to Vip3Aa, suggesting an early warning of resistance [15]. In addition, unexpected occurrence and damage of *H. zea* on Bt corn and cotton expressing Vip3Aa protein has been reported in some fields in the southern U.S. [13,14]. For example, *H. zea* larvae damaged approximately 67.5% Bt corn producing Cry1Ab, Cry1Fa, and Vip3Aa in a field trial in Snook, TX in 2018 [13]. Brown et al. [16] reported unexpected damage of *H. zea* to Bt cotton expressing Cry and Vip3Aa Bt proteins in two locations in Louisiana in 2018. Of greater concern, F_2_ screens with *H. zea* collected in Texas indicated that the frequency of major resistance alleles against Vip3Aa was not rare, with a value of 0.0065 during 2019 [17]. Moreover, corn and cotton producing Vip3Aa has recently gained increasing popularity among growers for control of *H. zea* because the efficacy of Cry proteins against *H. zea* has been largely compromised by the practical resistance in the field [14,18]. All these results suggest the risks of resistance to Vip3Aa in *H. zea* are increasing in the U.S. To ensure the success of the high-dose refuge strategy, the frequency of resistance allele in field insect populations is required to be low, ideally <0.001. Therefore, understanding the frequency of resistance alleles against Vip3Aa in *H. zea* populations is crucial for resistance management and the sustainability of Vip3Aa technology.

The traditional F_2_ screening method does not work well in determining resistance allele frequency for *H. zea* because the success of single-pair mating is very low [19,20,21]. To improve the efficiency of F_2_ screening for this pest, we recently developed a modified method by crossing three laboratory susceptible females with one feral male moth of *H. zea* to estimate its resistance allele frequency [22]. Using this method, we successfully screened 192 F_2_ families of *H. zea* against Cry1Ac and Cry2Ab2 Bt proteins with populations sampled from Arkansas, Louisiana, Mississippi, and Tennessee during 2019–2020 in a previous study [22]. We found the resistance allele frequency of *H. zea* in these four southern states for Cry1Ac and Cry2Ab was 0.217 and 0.722, respectively [22]. Here, we extended our study to report the resistance allele frequency against Vip3Aa of these 192 F_2_ families of *H. zea* collected from Arkansas, Louisiana, Mississippi, and Tennessee during 2019–2020. Among these 192 F_2_ families of *H. zea*, we found five families carrying major Vip3Aa resistance alleles. The estimated resistance allele frequency against Vip3Aa in *H. zea* for these four southern states is 0.0155, which is 15.5-fold relative to the desired value (<0.001) underlying the assumptions of the high-dose refuge strategy. These data should provide critical information for understanding the risks of Vip3Aa resistance in *H. zea* and help design appropriate resistance management strategies to increase the sustainability of the Vip3Aa technology for control of *H. zea* in the U.S.

## 2. Material and Methods

### 2.1. Insect Source and Establishment of H. zea F_2_ Families

A susceptible *H. zea* strain (SS) was obtained from Benzon Research Inc., Carlisle, PA in 2018. The SS strain was susceptible to Cry1Ac, Cry1A.105, Cry2Ab, and Vip3Aa proteins [23,24]. Feral larvae of *H. zea* were sampled from the fields in four southern states, Arkansas, Louisiana, Mississippi, and Tennessee, and the fresh larvae were shipped overnight to the Entomology Research Laboratory at Texas A&M University, College Station, TX during 2019–2020. Detailed collection data were listed in Table 1 as described in Santiago-González, et al. [22]. In addition, the percentage of corn acres planted containing Vip3Aa was 10.3–13.2% in Arkansas, 3.2–3.3% in Louisiana, 2.8–4.9% in Mississippi, and 7.7–11.3% in Tennessee during 2019–2020 [15]. The percentage of cotton acres planted containing Vip3Aa was 14.4–29.8% in Arkansas, 28.7–34.1% in Louisiana, 19.4–30.5% in Mississippi, and 25.4–33.9% in Tennessee during 2019–2020 [15]. The field larvae of *H. zea* were reared individually on meridic diet until the adult stage was reached as previously described in Yang et al. [13]. Each feral male *H. zea* was allowed to mate with three susceptible females (1 feral ♂ × 3 SS ♀) in a mating container to create F_1_ families. The containers were maintained at 26 ± 1 °C, ∼60% relative humidity (RH), and a photoperiod of 16:8 h (L:D) for 7 days. Offspring from the crossing of 1 feral ♂ × 3 SS ♀ was designated as an F_1_ family. Similarly, F_1_ insects were reared until the adult stage as previously described for those feral larvae. Sixty *H. zea* moths of each F_1_ family were sib-mated to produce the F_2_ generations. In general, a total of 52 and 140 F_2_ families of *H. zea* were established in 2019 and 2020, respectively.

### 2.2. Discriminating Concentration of F_2_ Screens

Neonates of *H. zea* from each F_2_ family were exposed to a discriminating concentration of 3.0 µg/cm^2^ Vip3Aa39 Bt protein in diet overlay bioassays as described before [17]. This discriminating concentration of 3.0 µg/cm^2^ Vip3Aa39 can kill 100% SS and Vip3Aa heterozygous *H. zea* insects, whereas the homozygous Vip3Aa resistant *H. zea* could survive well [25]. The Vip3Aa39 protein with concentrations of 0.9–2.9 mg/mL was provided by Dr. Juan Luis Jurat-Fuentes, University of Tennessee [17]. The bioassays were conducted using 128-well trays and each well was filled with 800 µL of liquid meridic corn earworm diet (Southland Product Inc., Lake Village, AR, USA). When the diet was solidified, a constant volume of 40 µL Vip3Aa39 protein solution (3.0 µg/cm^2^) suspended in 0.1% triton was overlaid onto the surface of each well of the bioassay trays. The solutions were allowed to air dry at room temperature, and then one neonate of *H. zea* (<24 h) was placed into each well using a damped fine painting brush. Wells were covered with air-vented lids, and the trays were maintained in the insectary for 7 days as described before [17]. The experiment consisted of 4 replicates with 32 neonates per replication (n = 4 × 32 = 128) for each F_2_ family and SS. The number of live larvae (molting to 2nd instar or above) was recorded after 7 days. In addition, insect survival and development on the control diet consisting of 40 µL 0.1% triton were also evaluated, and each field population of *H. zea* had a separate treatment of control in the bioassay.

### 2.3. Dose-Response Bioassays for Resistance Confirmation

In a total of 192 F_2_ families of *H. zea* evaluated in 2019–2020, the F_2_ bioassay identified five potential resistant families with 3rd and 4th instar survivors on the discriminatory concentration of 3.0 μg/cm^2^ Vip3Aa39 after 7 days. The survivors of each family were reared on the meridic diet and sib-mated to produce the F_3_ generation for resistance confirmation. Susceptibility to Vip3Aa39 protein of potential Vip3Aa resistant families and SS of *H. zea* was tested using a full range diet-overlay bioassay as described in Yang et al. [17]. The concentration of Vip3Aa39 used in the bioassay ranged from 0, 0.0316, 0.1, 0.316, 1.0, 3.16, 10.0, and 31.6 to 100 μg/cm^2^. Each combination of insect population and Vip3Aa39 concentration was replicated four times with 16 insects per replication. Bioassay trays were maintained at 26 ± 1 °C, 50% RH, and a 16:8 (L:D) h photoperiod. Larval mortality and development were recorded after 7 days of infestation.

### 2.4. Data Analysis

Survivorship for each *H. zea* F_2_ family at the discriminating Vip3Aa39 concentration was computed as 100 * number of surviving larvae at 2nd instar or above divided by the total insects assayed and the survivorship was then corrected according to the survival on the control treatment [26]. In this study, the resistant alleles identified in the F_2_ screen should originate from the feral males because the females were susceptible homozygotes. Theoretically in the F_2_ screen, the expected survival for F_2_ progeny in a family is 6.25% if the F_0_ feral male possesses a single recessive allele conferring the resistance and 25.0% if the F_0_ feral male possesses two recessive alleles conferring the resistance [27]. On the control diet, most larvae could develop to the 3rd or 4th instar. In this study, we defined a potential positive Vip3Aa resistant family as an F_2_ family that had survivors at least reaching the 3rd or 4th instar after 7 days on the discriminating concentration of Vip3Aa39 as described in Yang et al. [17].

Larval mortality in the full-range bioassays was calculated as the number of dead larvae plus those that were at the first instar divided by the total number of insects assayed. The mortality was also corrected based on the mortality of the control treatment [26]. Probit analysis was used to determine the median lethal concentration (LC_50_) and corresponding 95% fiducial limits (FL) using PROC PROBIT in SAS [28]. The LC_50_ of an *H. zea* population was considered greater than the highest Vip3Aa protein concentration used in the bioassay if larval mortality was <50% at the highest concentration. Resistance ratio was calculated using the LC_50_ value of an *H. zea* population divided by the LC_50_ of SS.

In a previous study, we used the high probability option of formula #3 in Andow et. al, [29] to calculate the frequency of alleles conferring resistance to Cry1Ac and Cry2Ab in *H. zea*, because the expected allele frequency of Cry1Ac and Cry2Ab is high in the field [22]. In this study, the Vip3A resistance allele frequency is expected to be low in the field [30]. Therefore, we calculated the frequency of alleles conferring resistance to Vip3Aa39 based on the small probability option of formula #3 in Andow et al. [29] with modifications for only a male F_2_ screen:

$$E\left[pR\right]=\frac{\left(S+u\right)}{2\left(N+u+v\right)}$$

where $E\;\left[pR\right]$ is the expected resistance allele frequency, S is the number of F_2_ families scored as resistant to Vip3Aa39 in the diagnostic screening, N is the total number of families screened, u  and v are parameters of the Beta probability distribution which varies from 0 to 1 [29]. The corresponding 95% credibility intervals were estimated according to Andow and Alstad [31]. The detection power of the F_2_ screen which is the efficacy that a resistance allele can be detected if it is present in a family line was calculated according to Stodola and Andow [32].

## 3. Results

### 3.1. Establishment of F_2_ Families and Survival in the Discriminating Concentration of Vip3Aa39

Similarly as described in Santiago-González et al. [22], 52 F_1_ families were established from a total of 212 crossings between feral *H. zea* males and SS females in 2019, and 140 F_1_ families were generated out of 411 crossings between feral *H. zea* males and SS females in 2020 (Table 1). All 192 F_1_ families were successfully sib-mated to produce sufficient F_2_ neonates for the F_2_ screening.

Survivorship of SS on the control diet was 93.7 ± 5.1%. However, 100% SS were killed at 3.0 μg/cm^2^ Vip3Aa39 protein, indicating again this discriminating concentration was high enough to identify Vip3Aa resistant insects in the F_2_ bioassays. In 2019, larval survival of the F_2_ insects on the control ranged from 87.5 to 96.1%, with a mean of 91.6 ± 1.7% (n = 5). A total of 6,656 insects from 52 families were assayed against Vip3Aa39, and three F_2_ families had survivors on 3.0 μg/cm^2^ Vip3Aa39 protein (Table 2). Two of them contained only 2nd instar larvae (Table 2). One family (LA-M1) from Alexandria, LA had four 3rd and seventeen 4th instar larvae (Table 3).

In 2020, the survivorship of the F_2_ insects on the control diet ranged from 71.1 to 97.7%, with a mean of 88.6 ± 2.2% (n = 12). About 17,920 neonates from 140 F_2_ families were assayed against the discriminating concentration of Vip3Aa39, and ten families contained survivors on the 3.0 μg/cm^2^ Vip3Aa39 protein (Table 2). Six of them contained only 2nd instar larvae (Table 2). One family (LA-AC4) from Winnsboro, LA had six 3rd and fourteen 4th instar larvae (Table 3). The remaining three families were from Stoneville, MS, with one family (MS-R21) containing one 4th instar, one family (MS-R2) containing one 2nd and one 4th instar, and one family (MS-R15) having three 2^nd^ and nineteen 3^rd^ instar larvae (Table 3). Based on the criteria of potential positive resistant families, we found five out of 192 F_2_ families of *H. zea* probably carrying at least one major Vip3Aa resistance allele during 2019–2020 (Table 3).

### 3.2. Dose-Response Bioassays for Resistance Confirmation

We successfully established four different populations out of the five potential Vip3Aa resistant families of *H. zea* identified in the F_2_ screen. The first population was LA-M1 which was established from collections of Cry1Ac+Cry2Ab2 cotton in Alexandria, LA in 2019. The second population was LA-AC4 that was originated from the collection of non-Bt corn in Winnsboro, LA in 2020. The remaining two populations were MS-R2 and MS-R15, both were from the collections of Cry1Ab corn in Stoneville, MS in 2020. All these four potential Vip3Aa resistant populations along with SS of *H. zea* were tested for susceptibility against Vip3Aa39 protein using the full range dose-response bioassays for resistance confirmation.

The LC_50_ of SS against Vip3Aa39 was estimated as 0.11 μg/cm^2^ with a 95% CL of 0.09–0.13 μg/cm^2^ (Table 4). Larvae from the four resistant populations were highly resistant to Vip3Aa39 protein and showed no differences (*p* > 0.05) in mortality (0–8.2%) across the populations and the tested concentrations. LC_50_ values for these four resistant populations could not be determined because the mortality at the highest tested concentration of 100.0 μg/cm^2^ was only 1.2–7.4%. Thus, the LC_50_ values for LA-M1, LA-AC4, MS-R2, and MS-R15 were all considered >100.0 μg/cm^2^, with an estimated resistance ratio >909.1-fold relative to SS (Table 4). These results suggested that all these four populations of *H. zea* are highly resistant to the Vip3Aa39 protein.

Based on the results of the F_2_ screen and dose-response confirmation bioassays, each of MS-R2 and MS-R21 collected from Stoneville, MS was presumed to carry one major resistance allele against Vip3Aa39 protein. LA-M1, LA-AC4, and MS-R15 contained 21, 20, and 22 survivors in the F_2_ screen, respectively (Table 3). Based on the average survival on the control diet, the corrected survivorship on Vip3Aa39 F_2_ screen for LA-M1, LA-AC4, and MS-R15 was 17.9, 17.6, and 19.4%, respectively. Moreover, inheritance studies showed the resistance to Vip3Aa39 protein in these three resistant Vip3Aa populations was recessive and controlled by a single gene (Yang et al., Unpublished data). The Chi-square (χ^2^) tests showed that the observed survival was not different (*p* > 0.05) from the expected survival at Vip3Aa39 concentration of 3.0 μg/cm^2^ if the F_0_ feral male possesses two recessive alleles conferring the resistance (Table 5). These results indicated that each of LA-M1, LA-AC4, and MS-R15 probably carried two major resistance alleles against Vip3Aa39 protein (Table 5).

Detailed expected frequency of Vip3Aa resistance alleles in *H. zea* by year and state are presented in Table 6. According to the overlapping of the 95% credibility intervals, the estimated resistance allele frequencies for Vip3Aa39 were not significantly different among states and years (Table 6). In 2019, two resistance alleles from 52 males were identified and the expected Vip3Aa resistance allele frequency was estimated as 0.0185 (CI 95%: 0.0023–0.0504) (Table 6). In 2020, six resistance alleles out of 140 male insects were found and the expected Vip3Aa resistance allele frequency was calculated as 0.0176 (CI 95%: 0.0058–0.0355) (Table 6). The pooled resistance allele frequency for Vip3Aa in *H. zea* collected from four southern states during 2019–2020 was estimated as 0.0155 with a 95% CI of 0.0057–0.0297) (Table 6). The detection power of the F_2_ screen in this study was estimated as 98.2%.

## 4. Discussion

The purpose of this study is to determine the frequency of alleles conferring resistance to Vip3Aa in *H. zea* in the mid-south region of the U.S. Using the modified F_2_ screen method by crossing susceptible laboratory female moths with a feral male moth of *H. zea*, we successfully screened a total of 24,576 neonates from 192 F_2_ families of *H. zea* collected in Arkansas, Louisiana, Mississippi, and Tennessee during 2019–2020. We found 2.6% F_2_ families contained ≥3rd instar survivors on the diagnostic concentration of Vip3Aa39. However, one limitation of this modified F_2_ screen method is that it only represents the genetics of male insects of the field population. In a previous study, Yang et al. [17] used female moths of *H. zea* collected from light traps to conduct the F_2_ screen against Vip3Aa39, and they observed 1.8% of 114 F_2_ families had ≥3rd instar survivors on the diagnostic concentration. In contrast, the light trap F_2_ screen method used only female insects of the field population, and these female moths could be fertilized by multiple male moths in the field. Considering the imperfection of each method, we recommend using both methods simultaneously for future Bt resistance monitoring of a field population of *H. zea* so that both male and female genetics can be fully understood.

In this study, we found five Vip3Aa resistant families through F_2_ screen and confirmed four of them possessed high levels of resistance using full range dose-response bioassays. Because we used the male insects collected from the field to mate with the laboratory susceptible female insects for the F_2_ screen, any resistant alleles identified in the study were from the feral male larvae. Based on the survival of F_2_ neonates in the bioassays and recessive inheritance data (Yang et al., Unpublished data), surprisingly, F_0_ feral larvae of LA-M1, LA-AC4, and MS-R15 were presumed to possess two recessive resistance alleles to Vip3Aa39, suggesting these *H. zea* larvae were homozygous resistant insects in the field. The first homozygous resistant larva (LA-M1) was collected from Cry1Ac+Cry2Ab2 cotton in Alexandria, LA in 2019; the second one (LA-AC4) was sampled from non-Bt corn in Winnsboro, LA in 2020; and the last one (MS-R15) was obtained from Cry1Ab sweet corn in Mississippi in 2020. Moreover, the other two families, MS-R2 and MS-R21 that were considered to have one recessive resistance allele to Vip3Aa39 were collected from the same field as MS-R15 in 2020. Thus three out of 16 larvae of *H. zea* tested from the Cry1Ab sweet corn field in Mississippi in 2020 contained Vip3Aa resistance alleles. On the contrary, no insects possessed Vip3Aa resistance alleles for the 89 F_2_ families of *H. zea* collected from Arkansas and Tennessee during 2019–2020. We did not observe any positive relationship between Vip3Aa resistance allele frequency and the percentage of corn and cotton containing Vip3Aa protein planted in these states during 2019–2020. For example, the percentage of corn planted containing Vip3Aa was 13.2% in Arkansas, 3.3% in Louisiana, 4.9% in Mississippi, and 11.3% in Tennessee in 2020. [15] The percentage of cotton planted containing Vip3Aa was 29.8% in Arkansas, 34.1% in Louisiana, 30.5% in Mississippi, and 33.9% in Tennessee in 2020. [15] The data showed more percentage of Vip3Aa corn and an equivalent percentage of Vip3Aa cotton were planted in Arkansas and Tennessee compared to that in Louisiana and Mississippi in 2020. On the contrary, more Vip3Aa resistance alleles were detected in Louisiana and Mississippi than that in Arkansas and Tennessee during 2020. In general, these results suggest that the resistance allele frequency of Vip3Aa in *H. zea* is high in Louisiana and Mississippi, which could explain the observation of unexpected occurrence and damage of *H. zea* on Bt corn and cotton expressing Vip3Aa proteins in these two states [15,16]. For example, 16 out of 200 randomly sampled Leptra corn ears (expressing Cry1Ab, Cry1F, and Vip3Aa) were damaged by *H. zea* larvae with an average of 5.5 damaged kernels per year in a field trial in Stoneville, MS in 2019 [15]. Brown et al. [16] showed unexpected occurrence and damage of *H. zea* to Bt cotton expressing Cry and Vip3Aa in two locations in Louisiana.

Yang et al. [17] reported that the frequency of major alleles conferring resistance to Vip3Aa39 in *H. zea* was 0.0065 (CI 95%: 0.0014–0.0157) in Texas in 2019. In the current study, we found the major resistance allele frequency for Vip3Aa in *H. zea* collected from four southern states during 2019–2020 was 0.0155 (CI 95%: 0.0057–0.0297). Based on the overlapping of the 95% confidence intervals, the estimated Vip3Aa resistance allele frequency was not significantly different between Texas and the four southern states. Contrary to the documentation of major Vip3Aa resistance alleles in these two studies, Lin et al. [33] found that none of the 101 F_2_ families of *H. zea* sampled from Louisiana, Mississippi, Georgia, and South Carolina possessed major alleles conferring resistance to Vip3Aa20. Lin et al. [33] used a group mating method by mixing multiple feral male and female moths of *H. zea* in a container to conduct F_2_ screens using a discriminating concentration of 5.0 µg/cm^2^ Vip3Aa20 during 2018–2019. Because *H. zea* females are polyandrous [34] and fitness costs are sometimes associated with Bt homozygous and/or heterozygous resistant insects, it is possible that *H. zea* without resistant alleles had a higher propensity to mate compared to those containing resistant alleles, which would result in underestimating the resistant allele frequencies in the population.

Data from the present study and previous studies suggest that resistance allele frequency for Vip3Aa in *H. zea* is not rare (<0.001) in Texas and the southeastern states [17,33]. However, field efficacy data indicated that Bt crops expressing Vip3Aa protein are still very effective for the management of *H. zea* in the field [30,35,36]. Currently, Vip3Aa protein is pyramided with Cry1 and/or Cry2 proteins in the commercialized Bt corn and Bt cotton products in the U.S. Many previous studies have indicated that strong cross-resistance was not present among Cry and Vip3Aa proteins [24,37,38,39]. In this study, we observed that all Vip3Aa resistant families of *H. zea* established in the F_2_ screen showed some levels of resistance to Cry1A with 2–13 3rd instar survivors (Table 7). However, all these Vip3Aa resistant families were susceptible to Cry2Ab2 with no 3rd instar survivors in the F_2_ screen (Table 7). In addition, full range diet-overlay bioassays of these Vip3Aa resistant families indicate that all these Vip3Aa resistant families show some low levels of resistance to Cry1Ac (resistance ratio <10) but are very susceptible to Cry2Ab2 based on the LC_50_ values relative to the SS, although Cry resistance in *H. zea* is extremely high in the field (Yang et al., Unpublished data). Additionally, previous studies have suggested that synergistic effects could be present in combinations of Cry and Vip3Aa proteins for some species. For example, Bergamasco et al. [40] found synergistic interaction between Cry1Ia and Vip3Aa proteins for *Spodoptera frugiperda* and *Spodoptera albula*. Soares Figueiredo et al. [41] also observed strong synergistic action for the combinations of Vip3Aa and Cry proteins against *S. frugiperda*. Baranek et al. [42] documented synergistic interactions between Cry1 and Vip3Aa proteins for *S. exigua.* All these factors could contribute to the high efficacy of Bt crops expressing both Cry and Vip3Aa proteins for control of *H. zea* in the field.

In a recent study, Dively et al. [43] used sentinel plots to monitor the susceptibility of *H. zea* against Cry and Vip3Aa proteins, and they found that field populations of *H. zea* not only showed high levels of resistance to Cry proteins but also decreased susceptibility against Vip3Aa. Although sweet corn expressing Vip3Aa could provide excellent control efficacy for *H. zea*, the number of living larvae and the proportion of larvae reaching the fourth instar on Vip3Aa expressing corn was significantly more than before [43]. In addition, protein bioassay data in the southern U.S. during 2016–2020 suggested an early warning of resistance to Vip3Aa in *H. zea* [15]. Furthermore, several studies have indicated that once the resistance allele frequency exceeds 0.005 in the field, the entire population can rapidly develop resistance [44,45]. Considering the frequency of Vip3Aa resistance alleles in *H. zea* in Texas and the southern states has surpassed this threshold, effective resistance management strategies, such as adopting incentives to promote refuge compliance and increasing refuge size, are strongly warranted to preserve the efficacy of Vip3Aa technology.

In a previous study, Yang et al. [25] characterized the Vip3A resistance in *H. zea* collected in Texas. In this study, we successfully established four different Vip3Aa-resistant populations of *H. zea* sampled from Louisiana and Mississippi. The availability of these resistant populations enables future research to understand the inheritance of Vip3Aa resistance, fitness costs, cross-resistance, and molecular mechanisms of Vip3Aa resistance among these different populations of *H. zea*. These types of information are essential for Bt resistance monitoring and developing effective resistance management programs to ensure the sustainability of Vip3Aa technology.

## Figures and Tables

**Table 1:** Success of establishing F_2_ families of *Helicoverpa zea* using a feral male moth mating with susceptible female moths.

Year	Collection Site	Host	Feral *H. zea* Males (♂) Collected for the Parental Cross	Number of Established F_2_ Families
2019	Alexandria, LA	Cry1A.105 + Cry2Ab2 corn	28	7
		Cry1Ac + Cry2Ab2 cotton	33	14
	Stoneville, MS	Cry1A.105 + Cry2Ab2 corn	30	5
	Leland, MS	Non-Bt host: soybean	32	10
	Jackson, TN	Non-Bt host: soybean	31	6
		Non-Bt host: sorghum	23	6
		Cry1Ab + Cry1F corn	35	4
Sub-total			212	52
2020	Stoneville, MS	Non-Bt host: corn	41	17
		Cry1Ab sweet corn	35	16
		Cry1A.105 + Cry2Ab2 corn	11	1
	Winnsboro, LA	Cry1A.105 + Cry2Ab2 corn	43	17
		Non-Bt host: corn	33	2
	Avoyelles, LA	Non-Bt host: soybean	37	8
	Alexandria, LA	Cry1A.105 + Cry2Ab2 corn	32	6
	Jackson, TN	Cry1A.105 + Cry2Ab2 corn	31	13
	Mississippi, AR	Cry1A.105 + Cry2Ab2 corn	63	28
		Non-Bt host: corn	42	16
	Pine Bluff, AR	Cry1A.105 + Cry2Ab2 corn	18	1
	Marianna, AR	Cry1A.105 + Cry2Ab2 corn	25	15
Sub-total			411	140
Total			623	192

**Table 2:** Survival of F_2_ families of *Helicoverpa zea* on the discriminating concentration of 3.0 µg/cm^2^ Vip3Aa39 after 7 days.

Year	Collection Site of the Feral Parental	No. Tested F_2_ Families	No. Surviving Families	No. 2nd Instar	No. 3rd Instar	No. 4th Instar
2019	Alexandria, LA	7	0	0	0	0
		14	1	0	4	17
	Stoneville, MS	5	0	0	0	0
	Leland, MS	10	2	2	0	0
	Jackson, TN	6	0	0	0	0
		6	0	0	0	0
		4	0	0	0	0
Sub-total		52	3	2	4	17
2020	Stoneville, MS	17	0	0	0	0
		16	3	4	19	2
		1	1	1	0	0
	Winnsboro, LA	17	1	1	0	0
		2	1	0	6	14
	Avoyelles, LA	8	1	1	0	0
	Alexandria, LA	6	1	1	0	0
	Jackson, TN	13	0	0	0	0
	Mississippi, AR	28	1	1	0	0
		16	1	1	0	0
	Pine Bluff, AR	1	0	0	0	0
	Marianna, AR	15	0	0	0	0
Sub-total		140	10	10	25	16
Total		192	13	12	29	33

**Table 3:** Potential resistant families containing survivors of *Helicoverpa zea* in the F_2_ screen on 3.0 μg/cm^2^ of Vip3Aa39 protein.

Family No.	No. Insects Screened	No. Survivors	No. Insect within Instar		
			2nd	3rd	4th
LA-M1	128	21	0	4	17
LA-AC4	128	20	0	6	14
MS-R2	128	2	1	0	1
MS-R15	128	22	3	19	0
MS-R21	128	1	0	0	1

**Table 4:** Dose-response bioassays of different *Helicoverpa zea* populations against Vip3Aa39 Bt protein.

Insect Strain	N ^a^	LC_50_ (95% CL) (µg/cm^2^) ^b^	Slope ± SE	χ^2^	df	Resistance Ratio ^c^
SS	512	0.11 (0.09, 0.13)	3.23 ± 0.35	12.9	26	-
LA-M1	512	>100	/	/	/	>909.1 *
LA-AC4	512	>100	/	/	/	>909.1 *
MS-R2	512	>100	/	/	/	>909.1 *
MS-R15	512	>100	/	/	/	>909.1 *

^a^ Total number of neonates assayed. ^b^ Larval mortality was calculated based on the number of dead larvae plus first instar survivors (mortality = dead + L1) divided by the total number of insects assayed. ^c^ Resistance ratio for a Bt protein was calculated by dividing the LC_50_ value of an insect population by that of the reference susceptible strain (SS). * Indicates highly significant resistance ratios (>10-fold).

**Table 5:** Test for feral male moths of *Helicovepa zea* containing two recessive resistance alleles in the F_2_ screen.

Insect Family	N ^#^	Observed Survival	Expected Survival *	χ^2^	*p-*Value
LA-M1	128	22.9	32	3.441	0.064
LA-AC4	128	22.5	32	3.738	0.054
MS-R15	128	24.8	32	2.141	0.143

^#^ Total number of neonates assayed. * Indicates the expected number of survivors in the F_2_ screen when the feral male moth of *H. zea* containing two recessive resistance alleles mating with the SS insects.

**Table 6:** Expected resistance allele frequency of *Helicoverpa zea* to Vip3Aa39.

Year	Collection Site of the Feral Parental	No. F_2_ Families Screened	No. Surviving Families	No. Resistance Alleles	Expected Resistance Allele Frequency	Credibility Interval (95%)
2019	Louisiana	21	1	2	0.0435	(0.0056–0.1142)
	Mississippi	15	0	0	0.0294	(0.0000–0.0854)
	Tennessee	16	0	0	0.0278	(0.0000–0.0808)
Sub-total		52	1	2	0.0185	(0.0023–0.0504)
2020	Louisiana	33	1	2	0.0286	(0.0036–0.0766)
	Mississippi	34	3	4	0.0556	(0.0160–0.1153)
	Tennessee	13	0	0	0.0333	(0.0000–0.0963)
	Arkansas	60	0	0	0.0081	(0.0000–0.0240)
Sub-total		140	4	6	0.0176	(0.0058–0.0355)
Total in two consecutive years		192	5	8	0.0155	(0.0057–0.0297)

**Table 7:** Survivors of Vip3Aa resistant families of *Helicoverpa zea* on Cry1Ac and Cry2Ab2 proteins in the F_2_ screen.

Insect Family	Cry1Ac Protein				Cry2Ab2 Protein			
	No. Insects Screened	No. Survivors			No. Insects Screened	No. Survivors		
		2nd	3rd	4th		2nd	3rd	4th
LA-M1	128	18	3	0	128	0	0	0
LA-AC4	128	7	10	0	128	0	0	0
MS-R2	128	30	11	0	128	8	0	0
MS-R15	128	18	13	0	128	2	0	0
MS-R21	128	21	2	0	128	1	0	0

## Data Availability

The data presented in this study are available on request from the corresponding author.
